# Multiple quantum filtered nuclear magnetic resonance of ^23^Na^+^ in uniformly stretched and compressed hydrogels

**DOI:** 10.1063/5.0158608

**Published:** 2023-07-18

**Authors:** S. J. Elliott, T. R. Eykyn, P. W. Kuchel

**Affiliations:** 1 Molecular Sciences Research Hub, Imperial College London, London W12 0BZ, United Kingdom; 2 School of Biomedical Engineering and Imaging Sciences, King’s College London, St Thomas’ Hospital, London SE1 7EH, United Kingdom; 3 School of Life and Environmental Sciences, University of Sydney, Sydney, NSW 2006, Australia

## Abstract

Stretching or compressing hydrogels creates anisotropic environments that lead to motionally averaged alignment of embedded guest quadrupolar nuclear spins such as ^23^Na^+^. These distorted hydrogels can elicit a residual quadrupolar coupling that gives an oscillation in the trajectories of single quantum coherences (SQCs) as a function of the evolution time during a spin-echo experiment. We present solutions to equations of motion derived with a Liouvillian superoperator approach, which encompass the coherent quadrupolar interaction in conjunction with relaxation, to give a full analytical description of the evolution trajectories of rank-1 (${\hat{T}}_{1\pm 1}$), rank-2 (${\hat{T}}_{2\pm 1}$), and rank-3 (${\hat{T}}_{3\pm 1}$) SQCs. We performed simultaneous numerical fitting of the experimental ^23^Na nuclear magnetic resonance (NMR) spectra and rank-2 (${\hat{T}}_{2\pm 1}$) and rank-3 (${\hat{T}}_{3\pm 1}$) SQC evolution trajectories measured in double and triple quantum filtered experiments, respectively. We estimated values of the quadrupolar coupling constant *C*_*Q*_, rotational correlation time *τ*_*C*_, and 3 × 3 Saupe order matrix. We performed simultaneous fitting of the analytical expressions to the experimental data to estimate values of the quadrupolar coupling frequency *ω*_*Q*_/2*π*, residual quadrupolar coupling $\left\langle \omega_{Q}\right\rangle /2\pi$, and corresponding spherical order parameter $S_{0}^{*}$, which showed a linear dependence on the extent of uniform hydrogel stretching and compression. The analytical expressions were completely concordant with the numerical approach. The insights gained here can be extended to more complicated (biological) systems such as ^23^Na^+^ bound to proteins or located inside and outside living cells in high-field NMR experiments and, by extension, to the anisotropic environments found *in vivo* with ^23^Na magnetic resonance imaging.

## I. INTRODUCTION

In ordered media, the intrinsic electric quadrupole moment of a guest nuclear spin *I* > 1/2 interacts with molecular-length-scale electric field gradients (EFGs) in the host milieu.^1^ Owing to the anisotropy of the medium, the coherent portion of the quadrupolar interaction is nonvanishing.^2^ This leads to a pronounced and well-resolved peak splitting in nuclear magnetic resonance (NMR) spectra of such samples.^3^ This effect has been studied in detail for the quadrupolar nuclear spins ^2^H, ^7^Li, ^9^Be, ^23^Na, and ^133^Cs in uniformly stretched hydrogels, which provide a means of creating uniform, reproducibly aligned environments that can mimic conditions in tissues and cells.^4^ The use of uniformly stretched hydrogels in NMR experiments has led to several informative applications: (*i*) quantification of ^13^C-labeled isotopomers and isotopologues in mixtures; (*ii*) discrimination of enantiomers in chiral molecules; (*iii*) molecular structure refinement; and (*iv*) analysis of molecular mobility.^5^

The ability to filter NMR signals via multiple quantum coherence (MQC) for nuclear spins *I* > 1/2 opens a range of ways to probe nuclear environments. The evolution of higher-rank single quantum coherences (SQCs) enables elucidation of both coherent and incoherent spin dynamics, such as estimates of residual quadrupolar couplings (RQCs) and relevant relaxation parameters, e.g., rotational correlation times of molecular tumbling, respectively. Simpson demonstrated that beat frequencies in the free induction decay (FID) of optically pumped ^201^Hg (*I* = 3/2) depend on sample orientation relative to the **B**_0_-field direction and symmetry of the containing vessel.^6^ Deschamps *et al.* observed similar orientation dependencies where the previously considered less-attractive (spectroscopically) isotope of ^131^Xe (*I* = 3/2) was employed to probe surfaces.^7^ Pavlovskaya and Meersmann filtered ^23^Na (*I* = 3/2) NMR signals via MQCs to spatially map flow-induced molecular alignment in biopolymer fluid with magnetic resonance imaging (MRI).^8^ Recently, lipid nanodiscs were employed as a magnetically aligned medium useful for the measurement of ^17^O (*I* = 5/2) RQCs of small molecules.^9,10^

In a biological context, free ^23^Na^+^ in solution typically resides in an isotropic environment with a short rotational correlation time (extreme narrowing regime). However, when transiently bound to proteins or membrane lipids, either inside or outside of a cell, free ^23^Na^+^ will adopt the much longer rotational correlation time of the larger entity (slow-motion regime). On the other hand, several biological environments give rise to anisotropic interactions. For example, the ordering found in skeletal and cardiac muscle suggests a means of discriminating extracellular ^23^Na^+^ from intracellular ^23^Na^+^ and hence provides a method of recording transmembrane ion flow *in vivo*.^11^ The membrane permeability^12^ and ionic dynamics^13^ of ^23^Na^+^ in cellular environments were probed by analyzing the relaxation characteristics of multiple-rank SQCs. ^23^Na^+^ molecular binding parameters of biomolecules in aqueous systems have also been determined via the same approach.^14^

In this article, we report a complete analysis of multiple quantum filtered (MQF) spectra and corresponding SQC evolution trajectories for ^23^Na^+^ embedded within uniformly stretched, compressed, and relaxed hydrogels. A distinct oscillation, observed at a frequency defined by the RQC, was reproducibly present in all ^23^Na SQC evolution trajectories from stretched and compressed hydrogels but was absent for a hydrogel in the relaxed state. The oscillating trajectories, including their overall sign, were well captured by both numerical simulations and solutions of equations of motion generated from operator evolution in Liouville space. Using these approaches, we were able to estimate quadrupolar coupling constants, residual quadrupolar couplings, and corresponding spherical order parameters, as well as rotational correlation times that were characteristic of these gel systems.

## II. THEORY

### A. Quadrupolar interaction

#### 1. Hamiltonian

In a uniformly anisotropic environment, the electric quadrupole moment of ^23^Na^+^ interacts with EFGs present at the nucleus. The governing quadrupolar Hamiltonian is:^15^

$${\hat{H}}_{Q}=\omega_{Q}\overset{+2}{\underset{m=-2}{\sum }}{\left(-1\right)}^{m}A_{2m}{\hat{T}}_{2-m},$$ (1)

where *ω*_*Q*_ is the quadrupolar coupling frequency of the ^23^Na nucleus:^16^

$$\omega_{Q}=\frac{3eQV_{zz}}{2I\left(2I-1\right)\hbar },$$ (2)

where *Q* is the electric quadrupole moment of the ^23^Na nucleus and *V*_*zz*_ is the EFG at the ^23^Na nucleus, which is the largest principal value of the EFG tensor (typically denoted *eq*). Note that the quadrupolar coupling constant, often defined as *C*_*Q*_ = *eQV*_*zz*_/*h*, is twice the value given in Eq. (2) for an *I* = 3/2 nuclear spin, i.e., *ω*_*Q*_/2*π* = *C*_*Q*_/2.

The quadrupolar Hamiltonian becomes time-dependent due to fluctuations in the amplitude of the EFG tensor and its orientation with respect to the uniform magnetic field **B**_0_. It is expedient to isolate the encoded time dependence by separating the quadrupolar Hamiltonian into a stationary component and a time-dependent perturbation:^17^

$${\hat{H}}_{Q}={\hat{H}}_{Q}^{0}+{\hat{H}}_{Q}^{1}\left(t\right),$$ (3)

where ${\hat{H}}_{Q}^{0}=\bar{{\hat{H}}_{Q}}$ is the time independent quadrupolar Hamiltonian responsible for the appearance of the NMR spectrum and ${\hat{H}}_{Q}^{1}\left(t\right)$ is the fluctuating quadrupolar Hamiltonian with $\bar{{\hat{H}}_{Q}^{1}\left(t\right)}=0$ that accounts for relaxation processes.

In high-field NMR, only diagonal elements of ${\hat{H}}_{Q}^{0}$ are considered with respect to the Zeeman Hamiltonian. Therefore, we obtain the secular Hamiltonian:^18^

$${\hat{H}}_{Q}^{0}=\omega_{Q}\langle A_{20}^{L}\rangle {\hat{T}}_{20},$$ (4)

where the angle brackets indicate averaging over molecular motion and the second-rank spherical tensor operator ${\hat{T}}_{20}$ is written in the laboratory frame (*L*):

$${\hat{T}}_{20}=\frac{3{\hat{I}}_{z}^{\;2}-I\left(I+1\right)\hat{I}}{\sqrt{6}},$$ (5)

where ${\hat{I}}_{z}$ is the *z*-angular momentum operator and $\hat{I}$ is the identity operator.

The central spatial spherical tensor element $\left\langle A_{20}^{L}\right\rangle$ is also written in the laboratory frame (*L*), which has a principal axis parallel to the **B**_0_-field direction, but the elements $A_{2m}^{P}$ are defined in the frame of the principal axis system (*P*):

$$A_{20}^{P}=\frac{1}{\sqrt{6}},$$ (6a)

$$A_{2\pm 1}^{P}=0,$$ (6b)

$$A_{2\pm 2}^{P}=\frac{\eta }{6},$$ (6c)

where *η* is the biaxiality of the EFG tensor and the principal axes of this frame coincide with those of the quadrupolar interaction. The orientation of the frame of the principal axis system (*P*) is fixed with respect to the molecular frame (*M*). The molecular frame (*M*) is fixed with respect to the ^23^Na^+^ ion and follows the rotational diffusion of the ^23^Na^+^ ion. Transformation of the spatial spherical tensor elements $A_{2m}^{P}$ from the frame of the principal axis system (*P*) to the molecular frame (*M*) is given by:

$$A_{2m^{\prime }}^{M}=\overset{+2}{\underset{m=-2}{\sum }}A_{2m}^{P}D_{mm^{\prime }}^{2}\left(\Omega_{PM}\right),$$ (7)

where $A_{2m}^{M}$ are the corresponding spatial spherical tensor elements written in the molecular frame (*M*) and the Wigner rotation matrix $D_{mm^{\prime }}^{2}\left(\Omega_{PM}\right)$^19^ brings both frames into coincidence with Euler angles $\Omega_{PM}=\left\{\alpha_{PM},\beta_{PM},\gamma_{PM}\right\}$.

The motionally averaged central spatial spherical tensor element written in the laboratory frame (*L*) is:

$$\langle A_{20}^{L}\rangle =\overset{+2}{\underset{m=-2}{\sum }}A_{2m}^{M}\langle D_{m0}^{2}\left(\Omega_{ML}\right)\rangle .$$ (8)

The motionally averaged elements of the Wigner rotation matrix $\left\langle D_{m0}^{2}\left(\Omega_{ML}\right)\right\rangle$ are the spherical order parameters of the ^23^Na^+^ ion:^20^

$$\langle D_{m0}^{2}\left(\Omega_{ML}\right)\rangle =S_{m}^{*},$$ (9)

with $S_{m}^{*}={\left(-1\right)}^{m}S_{-m}$. The spherical order parameters of the ^23^Na^+^ ion are described by an alignment tensor. The principal axes of the alignment tensor are assumed to be coincident with the axes of the molecular frame (*M*), with the alignment tensor diagonal in this frame.

The *zz*-element of the EFG tensor is a weighted average over all ^23^Na^+^ orientations. In a uniformly anisotropic medium, there is a preferred alignment of the EFG tensor with respect to the **B**_0_-field axis, and the molecular orientation dependent EFG is nonzero, i.e., ${\bar{V}}_{zz}\neq 0$. Consequently, the quadrupolar coupling frequency in Eq. (2) has a finite value. This ensemble average of the quadrupolar coupling frequency serves as a measure of the RQC interaction motionally averaged over all ^23^Na^+^ orientations.

The resonance line shape of the ^23^Na^+^ ions splits in the presence of a pronounced RQC, with a resolved frequency separation between the three equidistant peaks:

$$\langle \omega_{Q}\rangle =\sqrt{6}\;\omega_{Q}\left(A_{20}^{M}S_{0}^{*}+\left(A_{2+2}^{M}+A_{2-2}^{M}\right)S_{2}^{*}\right),$$ (10)

with:

$$S_{0}^{*}=\left\langle \frac{3\;Cos{\left(\beta_{\text{ML}}\right)}^{2}-1}{2}\right\rangle ,$$ (11a)

$$S_{\pm 2}^{*}=\left\langle \sqrt{\frac{3}{2}},\frac{Sin{\left(\beta_{\text{ML}}\right)}^{2}e^{\mp 2i\gamma_{\text{ML}}}}{2}\right\rangle ,$$ (11b)

where *β*_ML_ and *γ*_ML_ are two of the Euler angles describing the transformation of the spatial spherical tensor elements $A_{2m}^{M}$ from the molecular frame (*M*) to the laboratory frame (*L*).

#### 2. Relaxation

An explicit treatment of relaxation of quadrupolar nuclear spins in isotropic solution has been described in comprehensive detail elsewhere.^21^ In this case, the incoherent evolution of populations toward thermal equilibrium after perturbation is:^22^

$${\hat{\hat{\Gamma }}}_{Q}=-\overset{\infty }{\underset{0}{\int }}d\tau \;\bar{{\hat{\hat{H}}}_{Q}^{1}\left(t+\tau \right){\hat{\hat{H}}}_{Q}^{1}\left(t\right)}\;$$ (12a)

$$\begin{array}{ll} & \;=-\frac{1}{5}\omega_{Q}^{2}\overset{+2}{\underset{m^{\prime \prime },m^{\prime }=-2}{\sum }}A_{2m^{\prime \prime }}^{2}D_{m^{\prime \prime }m^{\prime }}^{2}\left(\Omega_{PM}\right)D_{m^{\prime \prime }m^{\prime }}^{2*}\left(\Omega_{PM}\right)\\ & \;\times \overset{+2}{\underset{m=-2}{\sum }}{\left(-1\right)}^{m}J\left(m\omega_{0}\right){\hat{\hat{T}}}_{2m}{\hat{\hat{T}}}_{2-m}, \end{array}$$ (12b)

where ${\hat{\hat{\Gamma }}}_{Q}$ is the quadrupolar relaxation superoperator and contains the double commutation superoperator of second-rank spherical tensor operators ${\hat{T}}_{2m}$. We note that a more sophisticated Lindbladian dissipator formalism has been presented for spin systems far from Boltzmann equilibrium.^23^ However, Eq. (12b) is valid for the high temperature approximation of a spin system at thermal equilibrium, which was sufficient to accurately reproduce our experimental results.

The spectral density function (that characterizes the spin bath of the lattice and is the essence of the Redfield theory of nuclear spin relaxation^24^) is the Fourier transform (FT) of the average autocorrelation function given in Eq. (12b). The spectral density function is described by:

$$J\left(m\omega_{0}\right)=\frac{\tau_{C}}{1+{\left(m\omega_{0}\tau_{C}\right)}^{2}},$$ (13)

where *ω*_0_ is the nuclear Larmor frequency and *τ*_*C*_ is the correlation time of overall rotational diffusion of the quadrupolar nuclear spins. The spectral density function is sampled at integer multiples *m* of *ω*_0_.

### B. Evolution

#### 1. Liouvillian

Quadrupolar nuclear spins undergo coherent interactions and are subject to resonant time-dependent fluctuations. Complete temporal evolution of quadrupolar nuclear spins is described by:^24^

$$\frac{d}{dt}\hat{\rho }\left(t\right)={\hat{\hat{L}}}_{Q}\hat{\rho }\left(t\right),$$ (14)

where $\hat{\rho }\left(t\right)$ is the spin density operator and ${\hat{\hat{L}}}_{Q}$ is the quadrupolar Liouvillian superoperator:

$${\hat{\hat{L}}}_{Q}=-i{\hat{\hat{H}}}_{Q}^{0}+{\hat{\hat{\Gamma }}}_{Q},$$ (15)

where ${\hat{\hat{H}}}_{Q}^{0}$ is the commutation superoperator of the time-averaged quadrupolar Hamiltonian.

In the quantum mechanical description of an MQF NMR experiment, the operator describing the initial *π*/2 excitation radio frequency (*rf*) pulse acts on the thermal equilibrium spin density operator (proportional to ${\hat{T}}_{10}$) to create rank-1 SQCs ${\hat{T}}_{1\pm 1}$. The delay periods *τ* correspond to free evolution under the quadrupolar interaction, which embrace a *π* *rf*-pulse to refocus chemical shifts and other interactions, see Fig. 2(a). As such, the total delay time *t* = 2*τ* and effective propagation of $\hat{\rho }\left(t\right)$ from time *t* = 0 is:

$$\hat{\rho }\left(t\right)=e^{{\hat{\hat{L}}}_{Q}\tau }e^{-i\pi {\hat{\hat{I}}}_{\phi }}e^{{\hat{\hat{L}}}_{Q}\tau }\hat{\rho }\left(0\right),$$ (16)

where $\hat{\rho }\left(0\right)={\hat{T}}_{1\pm 1}$ and *ϕ* is the phase of the refocusing *rf*-pulse. During the time intervals *τ*, the rank-1 SQCs ${\hat{T}}_{1\pm 1}$ evolve and partially convert into higher-rank SQCs, namely, ${\hat{T}}_{2\pm 1}$ and ${\hat{T}}_{3\pm 1}$, and the resulting spin density operator takes the form:

$$\hat{\rho }\left(t\right)=h_{11}\left(t\right){\hat{T}}_{1\pm 1}+h_{12}\left(t\right){\hat{T}}_{2\pm 1}+h_{13}\left(t\right){\hat{T}}_{3\pm 1},$$ (17)

where the functions *h*_1*l*_ are calculated as the expectation values of the corresponding spherical tensor operators ${\hat{T}}_{l\pm 1}$:

$$h_{1l}\left(t\right)=\mathrm{Tr}\left[\hat{\rho }\left(t\right){\hat{T}}_{l\pm 1}\right],$$ (18)

and describe the change in rank of spherical tensor operator from 1 to *l* = 1, 2, 3 that results from temporal dynamics under the quadrupolar interaction characterized by Eqs. (14)–(16).

#### 2. Solutions to equations of motion

In the following, the orientation of the principal axes of the frame of the principal axis system (*P*) are assumed to be coincident with those of the molecular frame (*M*) such that *β*_PM_ = *γ*_PM_ = 0. We assume that the EFG tensor of the quadrupolar nuclear spin is oriented with respect the **B**_0_-field axis such that $S_{0}^{*}$ is nonzero, i.e., $\beta_{\text{ML}}\neq ArcTan\left[\sqrt{2}\right]$. We also assume that there is no preferred alignment in the transverse plane, resulting in an average over *γ*_ML_ that leads to $S_{\pm 2}^{*}=0$. The EFG tensor was additionally assumed to be axially symmetric with a biaxiality of *η* = 0. In this way, the sole order parameter of relevance is $S_{0}^{*}$ with an RQC (splitting) of $\left\langle \omega_{Q}\right\rangle =\omega_{Q}S_{0}^{*}$. We use the nomenclature $h_{1l}\left(t\right)=g_{1l}\left(t\right)$ when *ω*_*Q*_/2*π* = 0 and $h_{1l}\left(t\right)=f_{\;1l}\left(t\right)$ when $\left\langle \omega_{Q}\right\rangle /2\pi =0$.

**Rank-1 SQCs** $\left({\hat{T}}_{1\pm 1}\right)$: The propagation of transverse magnetization described by the Liouville–von Neumann equation [Eq. (14)] involves coherent and incoherent influences that act collectively on the spin system. The time evolution of ${\hat{T}}_{1\pm 1}$ from the initial density operator $\hat{\rho }\left(0\right)={\hat{T}}_{1\pm 1}$ under the quadrupolar Liouvillian superoperator is:

$$\begin{array}{ll} h_{11}\left(t\right) & =\frac{2}{5}e^{-\frac{1}{5}\omega_{Q}^{2}\left(J\left(\omega_{0}\right)+J\left(2\omega_{0}\right)\right)t}+\frac{3}{5}e^{-\frac{1}{5}\omega_{Q}^{2}\left(J\left(0\right)+J\left(\omega_{0}\right)+J\left(2\omega_{0}\right)\right)t}\\ & \;\times \left(\cosh\left(\frac{1}{5}\sqrt{\omega_{Q}^{4}J{\left(2\omega_{0}\right)}^{2}-25{\left\langle \omega_{Q}\right\rangle }^{2}}t\right)\right.\\ & \left.\;+\frac{\omega_{Q}^{2}J\left(2\omega_{0}\right)}{\sqrt{\omega_{Q}^{4}J{\left(2\omega_{0}\right)}^{2}-25{\left\langle \omega_{Q}\right\rangle }^{2}}}\right.\\ & \left.\;\times \sinh\left(\frac{1}{5}\sqrt{\omega_{Q}^{4}J{\left(2\omega_{0}\right)}^{2}-25{\left\langle \omega_{Q}\right\rangle }^{2}}t\right)\right). \end{array}$$ (19)

In the absence of relaxation, and in the presence of a nonvanishing RQC, Eq. (19) reduces to:

$$g_{11}\left(t\right)=\frac{1}{5}\left(2+3\;\cos\;\langle \omega_{Q}\rangle t\right).$$ (20)

This simpler expression describes an oscillation at a beat frequency of $\left\langle \omega_{Q}\right\rangle /2\pi$ as a function of *t*, which is readily realized by making the simplification *ω*_*Q*_/2*π* = 0 Hz in Eq. (19).^25^ Alternatively, it is often the case in biological media that the three nuclear transition frequencies of ^23^Na^+^ are (nearly) degenerate.^26^ Under such circumstances, $\left\langle \omega_{Q}\right\rangle /2\pi =0$ Hz and ${\hat{T}}_{1\pm 1}$ evolves only under the influence of quadrupolar relaxation:^27^

$$f_{\;11}\left(t\right)=\frac{1}{5}\left(2e^{-\frac{1}{5}\omega_{Q}^{2}\left(J\left(\omega_{0}\right)+J\left(2\omega_{0}\right)\right)t}+3e^{-\frac{1}{5}\omega_{Q}^{2}\left(J\left(0\right)+J\left(\omega_{0}\right)\right)t}\right).$$ (21)

Under extreme narrowing conditions (fast motional limit) *ω*_0_*τ*_*C*_ ≪ 1 and $J\left(0\right)\approx J\left(\omega_{0}\right)\approx J\left(2\omega_{0}\right)$, therefore, Eq. (21) predicts monoexponential relaxation. However, in the slow-motion regime *ω*_0_*τ*_*C*_ ≫ 1 and $J\left(0\right)>J\left(\omega_{0}\right)>J\left(2\omega_{0}\right)$, Eq. (21) yields a biexponential decay function with fast and slow relaxation rates expected for quadrupolar nuclear spins *I* = 3/2.^27^

**Rank-2 SQCs** $\left({\hat{T}}_{2\pm 1}\right)$: The spherical tensor operator ${\hat{T}}_{1\pm 1}$ partially evolves into ${\hat{T}}_{2\pm 1}$ under the effects of the coherent quadrupolar interaction and incoherent relaxation phenomena. The time evolution of ${\hat{T}}_{2\pm 1}$ from the initial density operator $\hat{\rho }\left(0\right)={\hat{T}}_{1\pm 1}$ under the quadrupolar Liouvillian superoperator is:

$$\begin{array}{ll} h_{12}\left(t\right) & =\frac{i\sqrt{15}\langle \omega_{Q}\rangle e^{-\frac{1}{5}\omega_{Q}^{2}\left(J\left(0\right)+J\left(\omega_{0}\right)+J\left(2\omega_{0}\right)\right)t}}{\sqrt{\omega_{Q}^{4}J{\left(2\omega_{0}\right)}^{2}-25{\left\langle \omega_{Q}\right\rangle }^{2}}}\\ & \;\times \sinh\left(\frac{1}{5}\sqrt{\omega_{Q}^{4}J{\left(2\omega_{0}\right)}^{2}-25{\left\langle \omega_{Q}\right\rangle }^{2}}t\right). \end{array}$$ (22)

Note that the spherical tensor operator ${\hat{T}}_{2\pm 1}$ is a factor of *π*/2 out-of-phase relative to ${\hat{T}}_{1\pm 1}$. This change in tensor rank is solely attributable to the coherent portion of the quadrupolar interaction that transforms tensors between odd and even ranks;^28^ i.e., for isotropic environments where $\left\langle \omega_{Q}\right\rangle /2\pi =0$ Hz, then $f_{\;12}\left(t\right)=0$. In the absence of relaxation, and in the presence of a nonvanishing RQC, Eq. (22) simplifies to:

$$g_{12}\left(t\right)=i\sqrt{\frac{3}{5}}\sin\left(\langle \omega_{Q}\rangle t\right).$$ (23)

A beat frequency of $\left\langle \omega_{Q}\right\rangle /2\pi$ is also predicted as a function of *t* in the case of coherent quadrupolar evolution in the presence of nonzero RQCs.

**Rank-3 SQCs** $\left({\hat{T}}_{3\pm 1}\right)$: *I* = 3/2 nuclear spins permit evolution of ${\hat{T}}_{1\pm 1}$ into rank-3 SQCs, which evolve in a similar manner to transverse magnetization. The solution of the equation of motion for the generation of the spherical tensor operator ${\hat{T}}_{3\pm 1}$ from the initial density operator $\hat{\rho }\left(0\right)={\hat{T}}_{1\pm 1}$ under the quadrupolar Liouvillian superoperator is:

$$\begin{array}{ll} h_{13}\left(t\right) & =-\frac{\sqrt{6}}{5}e^{-\frac{1}{5}\omega_{Q}^{2}\left(J\left(\omega_{0}\right)+J\left(2\omega_{0}\right)\right)t}+\frac{\sqrt{6}}{5}e^{-\frac{1}{5}\omega_{Q}^{2}\left(J\left(0\right)+J\left(\omega_{0}\right)+J\left(2\omega_{0}\right)\right)t}\\ & \;\times \left(\cosh\left(\frac{1}{5}\sqrt{\omega_{Q}^{4}J{\left(2\omega_{0}\right)}^{2}-25{\left\langle \omega_{Q}\right\rangle }^{2}}t\right)\right.\\ & \left.\;+\frac{\omega_{Q}^{2}J\left(2\omega_{0}\right)}{\sqrt{\omega_{Q}^{4}J{\left(2\omega_{0}\right)}^{2}-25{\left\langle \omega_{Q}\right\rangle }^{2}}}\right.\\ & \left.\;\times \sinh\left(\frac{1}{5}\sqrt{\omega_{Q}^{4}J{\left(2\omega_{0}\right)}^{2}-25{\left\langle \omega_{Q}\right\rangle }^{2}}t\right)\right). \end{array}$$ (24)

In the absence of relaxation, and in the presence of a nonvanishing RQC, Eq. (24) becomes:^25^

$$g_{13}\left(t\right)=\frac{\sqrt{6}}{5}\left(\cos\;\langle \omega_{Q}\rangle t-1\right),$$ (25)

where the spherical tensor operator ${\hat{T}}_{3\pm 1}$ created is a factor of *π* out-of-phase with ${\hat{T}}_{1\pm 1}$. An oscillation at a beat frequency of $\left\langle \omega_{Q}\right\rangle /2\pi$ as a function of *t* is again apparent if an RQC is present and at least partially observable (resolved), e.g., due to an anisotropic environment, which is evident if *ω*_*Q*_/2*π* = 0 Hz in Eq. (24).^25^ If quadrupolar relaxation is active, and RQCs are (close to) zero or not readily observable (resolved), e.g., in isotropic environments where $\left\langle \omega_{Q}\right\rangle /2\pi =0$, the solution of the equation of motion for rank-3 SQC evolution becomes:

$$f_{\;13}\left(t\right)=\frac{\sqrt{6}}{5}\left(e^{-\frac{1}{5}\omega_{Q}^{2}\left(J\left(0\right)+J\left(\omega_{0}\right)\right)t}-e^{-\frac{1}{5}\omega_{Q}^{2}\left(J\left(\omega_{0}\right)+J\left(2\omega_{0}\right)\right)t}\right),$$ (26)

in agreement with the literature.^27^ Under extreme narrowing conditions, *ω*_0_*τ*_*C*_ ≪ 1 and $J\left(0\right)\approx J\left(\omega_{0}\right)\approx J\left(2\omega_{0}\right)$, then $f_{\;13}\left(t\right)=0$. However, in the slow-motion regime, *ω*_0_*τ*_*C*_ ≫ 1 and $J\left(0\right)>J\left(\omega_{0}\right)>J\left(2\omega_{0}\right)$, Eq. (26) again yields a characteristic biexponential buildup and decline function with fast and slow rate constants for quadrupolar nuclear spins *I* = 3/2. ${\hat{T}}_{3\pm 1}$ can therefore be observed in triple quantum filtered (TQF) experiments in isotropic environments, provided rotational reorientation is slow on the NMR timescale.

#### 3. Motional regimes

Equations (19), (22), and (24) are complete analytical descriptions of the evolution trajectories for coherences of rank-1 ${\hat{T}}_{1\pm 1}$, rank-2 ${\hat{T}}_{2\pm 1}$, and rank-3 ${\hat{T}}_{3\pm 1}$, respectively. Similar analytical expressions have been previously reported for ^131^Xe nuclear spins interacting with surfaces.^7^ However, to the best of our knowledge, full analytical expressions have not been presented for ^23^Na^+^ embedded within mechanically aligned hydrogels.

We have verified that the analytical expressions agree with the numerical integration of Eq. (14); see Fig. S1 of the supplementary material. For a set of typical parameters, the evaluated solutions of the equations of motion were indistinguishable from the numerical results.

**FIG. 1.**
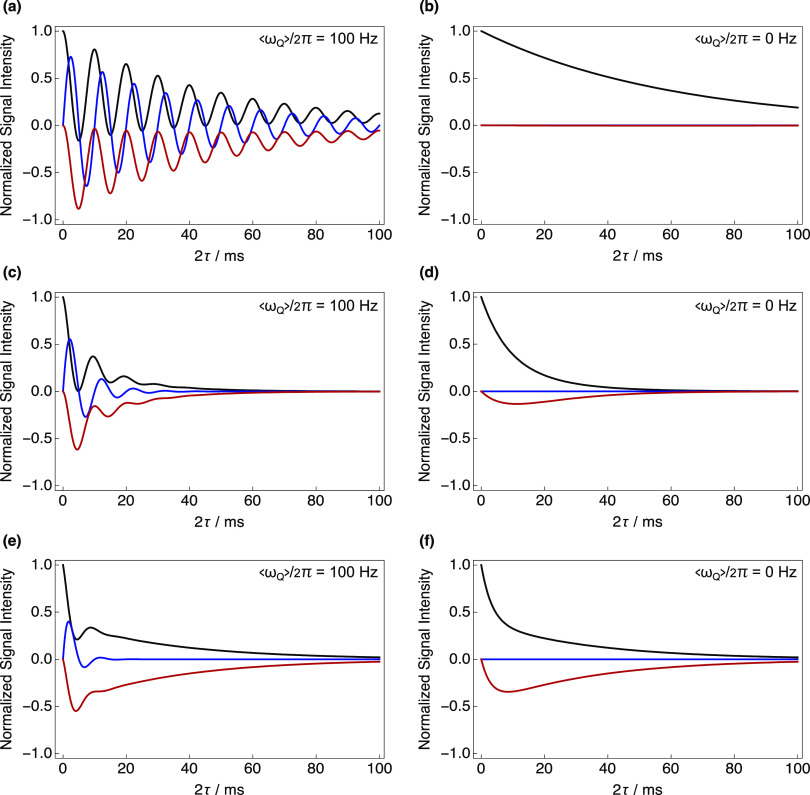
Evaluation of the analytical expressions for the SQC trajectories of rank-1 *h*_11_(*t*) (black), rank-2 *h*_12_(*t*)/*i* (blue), and rank-3 *h*_13_(*t*) (red) terms. Evolution functions were calculated with *ω*_*Q*_/2*π* = 84.5 kHz for the (a), (b) fast motion regime: *τ*_*C*_ = 1.5 × 10^−10^ s; (c), (d) intermediate motion regime: *τ*_*C*_ = 1.5 × 10^−9^ s; and (e), (f) slow-motion regime: *τ*_*C*_ = 5 × 10^−9^ s. The left column corresponds to anisotropic environments with ⟨*ω*_*Q*_⟩/2*π* = 100 Hz, while the right column corresponds to isotropic environments with ⟨*ω*_*Q*_⟩/2*π* = 0 Hz.

The functional forms of these derived analytical expressions were examined under various motional regimes reported in the literature.^26–28^ Figures 1(a) and 1(b) show calculations with analytical expressions *h*_11_(*t*), *h*_12_(*t*), and *h*_13_(*t*) in Eqs. (19), (22), and (24), respectively, in the extreme narrowing limit with a short rotational correlation time of *τ*_*C*_ = 1.5 × 10^−10^ s and an RQC of (a) $\left\langle \omega_{Q}\right\rangle /2\pi =100$ Hz and (b) $\left\langle \omega_{Q}\right\rangle /2\pi =0$ Hz. The SQC evolution trajectories shown in Fig. 1(a) show a pronounced oscillation with a beat frequency of $\left\langle \omega_{Q}\right\rangle /2\pi$, according to Eqs. (20), (23) and (25). In Fig. 1(b), which corresponds to a fast, isotropic motional regime, a monoexponential decay of *h*_11_(*t*) is clearly observed, while the terms *h*_12_(*t*) and *h*_13_(*t*) both vanished.

Figures 1(c) and 1(d) show evaluations of the functions *h*_11_(*t*), *h*_12_(*t*), and *h*_13_(*t*) in an intermediate motional regime with a rotational correlation time of *τ*_*C*_ = 1.5 × 10^−9^ s and an RQC of (c) $\left\langle \omega_{Q}\right\rangle /2\pi =100$ Hz and (d) $\left\langle \omega_{Q}\right\rangle /2\pi =0$. The evolution trajectories in Fig. 1(c) display a pronounced oscillation at the same beat frequency as in Fig. 1(a), but the decay is dampened more rapidly because nuclear spin–spin relaxation is faster due to slower reorientation. In Fig. 1(d), corresponding to intermediate isotropic motion, the decay of *h*_11_(*t*) becomes biexponential, the term *h*_12_(*t*) vanishes, and *h*_13_(*t*) displays the characteristic biexponential buildup and decline.

Figures 1(e) and 1(f) show the evaluated functions *h*_11_(*t*), *h*_12_(*t*), and *h*_13_(*t*) in a slow motional regime, such as when ^23^Na^+^ is bound to a protein, with a rotational correlation time of *τ*_*C*_ = 5 × 10^−9^ s and an RQC of (e) $\left\langle \omega_{Q}\right\rangle /2\pi =100$ Hz and (f) $\left\langle \omega_{Q}\right\rangle /2\pi =0$ Hz. The evolution trajectories in Fig. 1(e) again display an oscillation at the same beat frequency but are much less pronounced than before. The theoretical curves display much more pronounced biexponential nuclear spin–spin relaxation, with fast and slow components. In Fig. 1(f), corresponding to slow isotropic motion, the biexponential decay of *h*_11_(*t*) is readily observed, *h*_12_(*t*) again vanishes, and the term *h*_13_(*t*) again displays the characteristic biexponential buildup and decline.

Several key features are evident from the evolution trajectories shown in Fig. 1. Rank-2 coherence is only produced in the presence of a nonzero RQC. Under isotropic conditions, where $\left\langle \omega_{Q}\right\rangle /2\pi =0$, the rank-2 coherence always vanishes regardless of the motional regime. As predicted from Eq. (26), under extreme narrowing conditions, the rank-3 coherence also vanishes, while under intermediate or slow rotational reorientation the rank-3 coherence displays a characteristic biexponential buildup and decline. These observations form the basis of multiple quantum filtering of different SQCs to yield ^23^Na NMR signals that are sensitive to different motional regimes in isotropic or anisotropic environments.

## III. EXPERIMENTS

### A. Sample preparation

For 35% *w*/*v* hydrogels, 3.5 g of granulated bovine gelatin (*Gelita*, Brisbane, Queensland, Australia) was suspended in 10 ml of 60 mM NaOH/120 mM NaCl in a 50 ml disposable plastic centrifuge tube. For 60% *w*/*v* hydrogels, the medium was 103 mM NaOH and 350 mM NaCl. Physiological pH (pH = 7.2–7.4) was only achieved by these surprisingly high concentrations of NaOH. The gelatin solution was heated to 80 °C for ∼30 min and centrifuged at ∼2000 × g for 20 s to remove air bubbles. The gelatin solution was drawn into a 25 cm long silicone rubber tube (*Sims Portex*, Hythe, Kent, United Kingdom; 7 mm o.d., 5 mm i.d.) via an attached 10 ml plastic syringe. The end of the tube was then sealed with a Delrin plug, taking care to avoid introducing air bubbles. The loaded silicone rubber tube was inserted into the bore of a bottomless thick-walled glass NMR tube (*New Era*, Vineland, New Jersey, United States of America). For samples that were subsequently compressed, the silicone tube was stretched by 15 cm, up to a maximum of 20 cm, and held in that state by a custom-made nylon thumbscrew that rested on the top of the glass tube. Once the gelatin had set on being cooled to 20 °C, the stretched silicone tube was slowly released to compress the contents. For stretched samples, these were cooled below 20 °C before stretching in the device. The samples were lowered into the bore of the NMR magnet on a plumbers’ line as the air-ejector could not support the weight of the stretching/compression assembly. Using the above approach, we prepared a range of hydrogels under varying degrees of uniform compression (−7.5 and −15 cm) and stretching (5, 10, 15 cm) of a 20 cm sample as well as in the relaxed state (0 cm).

### B. Pulse sequences

**FIG. 2.**
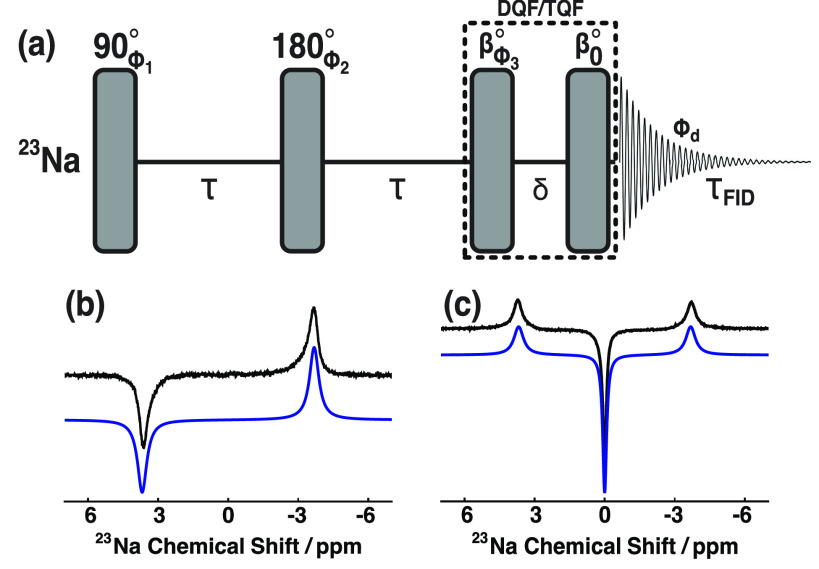
(a) Radio frequency pulse sequence used for creating, filtering, and detecting SQCs of ^23^Na^+^ in uniformly stretched and compressed hydrogels. (b) DQF and (c) TQF ^23^Na spectra. Black solid line: experimental spectra; blue solid line: simulated spectra. Spectra were simulated using the *Mathematica*-based NMR software package *SpinDynamica*. Simulation parameters: $S_{0}^{*}$ = 4.6 × 10^−3^, *ω*_*Q*_/2*π* = 84.5 kHz, and *τ*_*C*_ = 1.72 ns.

The *rf*-pulse sequence used to prepare, filter, and observe SQCs of ^23^Na^+^ in hydrogels is shown in Fig. 2(a). The MQF *rf*-pulse sequence works as follows: (*i*) a preparatory *π*/2 *rf*-pulse creates transverse magnetization from longitudinal order; (*ii*) the SQC ${\hat{T}}_{1\pm 1}$ partially evolves into second- and third-rank SQCs ${\hat{T}}_{2\pm 1}$ and ${\hat{T}}_{3\pm 1}$ according to Eqs. (22) and (24), respectively; (*iii*) the *π* *rf*-pulse in the middle of the evolution period 2*τ* refocuses the chemical shift interaction, off-resonance effects, nonuniformities in magnetic susceptibility, and **B**_0_-field inhomogeneities; (*iv*) the third *rf*-pulse, in conjunction with a suitable *rf*-pulse nutation angle *β* and phase cycling of the first three *rf*-pulses, selects the desired rank of MQC, i.e., signal filtration via relevant MQC, and removes unwanted terms generated from spurious magnetization; (*v*) the non-observable second- and third-rank double or triple quantum coherences ${\hat{T}}_{2\pm 2}$ or ${\hat{T}}_{3\pm 3}$, respectively, are converted into observable magnetization by the final *rf*-pulse after a short delay (typically *δ* = 40 *μ*s); (*vi*) an FID is detected; and (*vii*) FT of this signal yields the corresponding spectrum. Step (*ii*) is explained in more detail in Sec. II B 1. Spin-echo experiments did not employ the last two *rf*-pulses, which correspond to the MQF. Spin-echo experiments to observe ${\hat{T}}_{1\pm 1}$ were acquired with a two-step phase cycle: *ϕ*_1_ = {90°, 270°}, *ϕ*_2_ = {0°, 0°}, *ϕ*_d_ = {0°, 180°}. Double quantum filtered (DQF) experiments to observe ${\hat{T}}_{2\pm 1}$ were acquired with a four-step phase cycle: *ϕ*_1_ = *ϕ*_2_ = {90°, 180°, 270°, 0°}, *ϕ*_3_ = {0°}, *ϕ*_d_ = {0°, 180°, 0°, 180°} with the *rf*-pulse flip angle *β* set to the magic angle, i.e., 54.7°. The use of a magic angle *rf*-pulse ensures that rank-3 double quantum coherence ${\hat{T}}_{3\pm 2}$ is not observed. TQF experiments to observe ${\hat{T}}_{3\pm 1}$ were acquired with a six-step phase cycle: *ϕ*_1_ = {30°, 90°, 150°, 210°, 270°, 330°}, *ϕ*_2_ = {120°, 180°, 240°, 300°, 0°, 60°}, *ϕ*_3_ = {0°}, *ϕ*_d_ = {0°, 180°, 0°, 180°, 0°, 180°} with the *rf*-pulse flip angle *β* = 90°.

### C. Data acquisition and processing

All experiments were performed on a *Bruker Biospin* 9.4 T NMR magnet (^23^Na nuclear Larmor frequency = 105.93 MHz) at 20 °C. Experimental ^23^Na spectra were acquired with a spectral width of 40 ppm, 2048 time domain data points, and a corresponding acquisition time of 0.24 s. NMR and MQF spectra were acquired with 8 and 96 transients, respectively. An interval of 0.2 s was used between successive transients. An exponential line broadening factor of 0.3 Hz was applied prior to FT and subsequent baseline correction.

To achieve experimental ^23^Na rank-2 and rank-3 SQC evolution trajectories as a function of the echo delay 2*τ*, a series of DQF and TQF spectra were acquired in a pseudo-2D fashion using variants of the *rf*-pulse sequence shown in Fig. 2(a) with a variable delay *τ* and 128 timepoints. The experimental rank-2 and rank-3 SQC evolution trajectories were calculated by integrating the low frequency outer transition of the DQF spectrum or the central transition of the TQF spectrum, respectively. Peak integrals were plotted as a function of 2*τ* and normalized with respect to the most intense data point. Figures 2(b) and 2(c) show representative ^23^Na DQF and TQF spectra, respectively, at an optimum value of *τ* for the case of a nonzero RQC. The characteristic line shapes can be understood by taking the FT of Eqs. (23) and (25).^29^

### D. Numerical and analytical fitting

Experimental ^23^Na NMR data were simulated using a numerical procedure from the *MatLab*-based NMR software package *Spinach*,^30^ see Fig. S2 of the supplementary material. Simultaneous fitting was achieved by stitching together the experimental data, composed of the normalized ^23^Na NMR spectrum and rank-2 and rank-3 SQC evolution trajectories, into a single vector of data points. Simulated data of the same form were calculated numerically. Least squares fitting was performed by minimizing an error function corresponding to the square of the difference between the normalized experimental and simulated vectors. The Saupe order matrix was given by the function inter.order_matrix = A × diag([−0.5 −0.5 1.0]), where A is a scaling factor included as a fitting parameter. The Euler angle *β*_ML_ in Eq. (11a) was set to 0 or *π*/2 for all uniformly stretched or compressed hydrogels, respectively, so that the sign of $\left\langle \omega_{Q}\right\rangle /2\pi$ was inverted. This was equivalent to $S_{0}^{*}$ allowing for variable degrees of uniform stretching (A > 0) and compression (A < 0) or for the relaxed state (A = 0). Using the above procedure led to an estimate of the 3 × 3 order matrix elements and best fit parameters *C*_*Q*_ and *τ*_*C*_. $S_{0}^{*}$ was calculated from the principal component of the alignment tensor multiplied by a factor of $\left(3\;Cos{\left(\beta_{\text{ML}}\right)}^{2}-1\right)/2$.

The diagonal order matrix and best fit parameters derived from *Spinach* were subsequently used as initial values to simultaneously fit the theoretical expressions derived in Sec. II B 1 to the same experimental ^23^Na rank-2 and rank-3 SQC evolution trajectories, see Fig. S3 of the supplementary material. Simultaneous fitting of the two analytical expressions given by Eqs. (22) and (24) was achieved by stitching together the experimental data, composed of the rank-2 and rank-3 SQC evolution trajectories, into a single vector of data points. The analytical rank-2 and rank-3 SQC evolution trajectories were likewise calculated. Least squares fitting was performed by minimizing an error function corresponding to the square of the difference between the experimental and calculated data. This procedure derived a single set of best fit parameter values for $\left\langle \omega_{Q}\right\rangle /2\pi$, *ω*_*Q*_/2*π*, and *τ*_*C*_, from which $S_{0}^{*}=\left\langle \omega_{Q}\right\rangle /\omega_{Q}$ was calculated for each sample.

## IV. RESULTS

### A. Numerical fitting

Numerical least squares fitting produced good fits to the experimental ^23^Na NMR spectra and rank-2 and rank-3 SQC evolution trajectories, see Fig. S2 of the supplementary material. The mean quadrupolar coupling constant and average rotational correlation time across all hydrogel samples were found to be *C*_*Q*_ = 167 ± 7 kHz and *τ*_*C*_ = 1.5 ± 0.2 ns, respectively. Values returned from the numerical fitting procedure are given in Table S1 of the supplementary material.

### B. ^23^Na NMR spectra

**FIG. 3.**
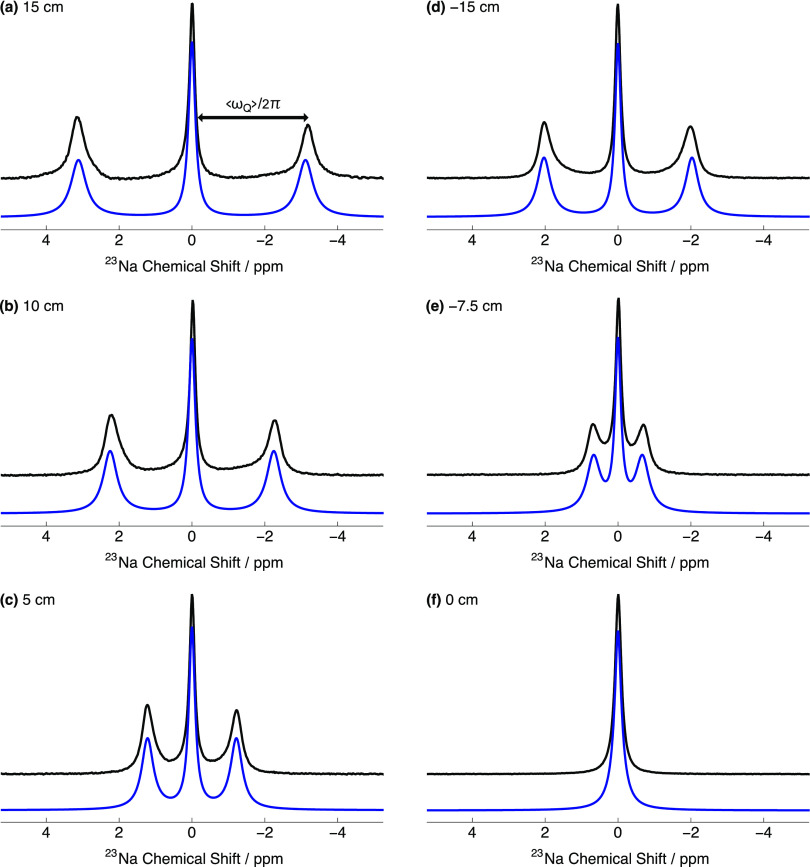
Relevant portions of the experimental NMR spectra of ^23^Na^+^ in a hydrogel sample acquired under several conditions of uniform stretching and compression and in the relaxed state. Stretching extent: (a) 15 cm; (b) 10 cm; and (c) 5 cm. Compression extent: (d) 15 cm; and (e) 7.5 cm. (f) Relaxed state. Black solid line: experimental spectrum; blue solid line: simulated spectrum. Spectra were simulated using the *Mathematica*-based NMR software package *SpinDynamica*.

Figure 3 shows the relevant portions of the experimental and simulated NMR spectra of ^23^Na^+^ in uniformly stretched, compressed, and relaxed hydrogels. Numerical simulations were performed using Eqs. (14) and (15) and the parameters derived from the numerical fitting procedure, which gave good fits to the experimental ^23^Na NMR spectra. Fitting values returned for all ^23^Na NMR spectra are reported in the supplementary material.

In the case of a sample uniformly stretched to an extent of 15 cm, the 3:4:3 multiplet structure shown in Fig. 3(a) has three well-resolved peaks separated by an RQC of $\left\langle \omega_{Q}\right\rangle /2\pi =330.3$ Hz. The experimental ^23^Na NMR spectrum in Fig. 3(a) was well simulated using the following parameters: $S_{0}^{*}$ = 3.77 × 10^−3^, *ω*_*Q*_/2*π* = 87.6 kHz, and *τ*_*C*_ = 1.56 ns.

Figures 3(b) and 3(c) show experimental ^23^Na NMR spectra of a hydrogel uniformly stretched to lesser extents. The RQC was diminished in these spectra due to the reduced alignment of ^23^Na^+^ within the gelatin sample. The simulations likewise give a good fit to these experimental ^23^Na NMR spectra.

Figures 3(d) and 3(e) show experimental ^23^Na NMR spectra for a hydrogel sample uniformly compressed to different extents. These spectra are similar in appearance to those in Figs. 3(a)–3(c), i.e., a 3:4:3 multiplet structure is again observed (only partial resolved in the case of a compression extent of −7.5 cm). Note that the value of the RQC is smaller under compression vs the case of uniform hydrogel stretching to the same extent. For example, the RQC in Fig. 3(d) is $\left\langle \omega_{Q}\right\rangle$/2*π* = −215.2 Hz. This is due to the preference for alignment of ^23^Na^+^ perpendicular to the **B**_0_-field direction in these gels. The experimental ^23^Na NMR spectra were well simulated using the parameters derived from the numerical fitting procedure. In the case of Fig. 3(d), the parameters derived from the numerical fitting procedure were: $S_{0}^{*}=$ −2.64 × 10^−3^, *ω*_*Q*_/2*π* = 81.5 kHz, and *τ*_*C*_ = 1.48 ns.

The splitting vanishes in the relaxed state (0 cm of gel stretch or compression) where there is no alignment of the ^23^Na^+^ in the hydrogel matrix, as seen in Fig. 3(f). The 3:4:3 multiplet structure previously observed now collapses to a singlet in this isotropic system with an RQC of $\left\langle \omega_{Q}\right\rangle /2\pi =$ 0 Hz. The experimental ^23^Na NMR spectrum was well simulated using the parameters derived from the numerical fitting procedure: $S_{0}^{*}=$ 0.0 × 10^−3^, *ω*_*Q*_/2*π* = 77.5 kHz, and *τ*_*C*_ = 1.70 ns.

### C. ^23^Na longitudinal relaxation

We also measured the longitudinal nuclear spin relaxation time constants *T*_1_ of the central and outer ^23^Na NMR peaks using the inversion recovery experiment. Interestingly, the spectrum showed differential *T*_1_ relaxation in stretched and compressed samples. We found the inner peak to have a shorter value of *T*_1_ = 20.1 ± 0.1 ms, while for the outer peaks longitudinal relaxation time constants were longer with *T*_1_ = 20.8 ± 0.4 ms and *T*_1_ = 20.7 ± 0.3 ms (average values of *T*_1_ quoted).

### D. Analytical fitting

Analytical least squares fitting to the complete solutions of the equations of motion for rank-2 and rank-3 SQC evolution derived in Sec. II B 1 produced good fits to the experimental ^23^Na trajectories, see Fig. S3 of the supplementary material. The mean quadrupolar coupling and average rotational correlation time across all hydrogel samples were found to be in good agreement with those achieved via the numerical fitting procedure: *ω*_*Q*_/2*π* = 80 ± 2 kHz and *τ*_*C*_ = 1.6 ± 0.2 ns. The RQC varied across hydrogel samples from 328.9 Hz (stretching extent = 15 cm) to −217.5 Hz (compression extent = 15 cm) again in good agreement with the numerical fitting approach. Values obtained from the analytical fitting method are reported in Table S2 of the supplementary material.

### E. ^23^Na SQC evolution trajectories

**FIG. 4.**
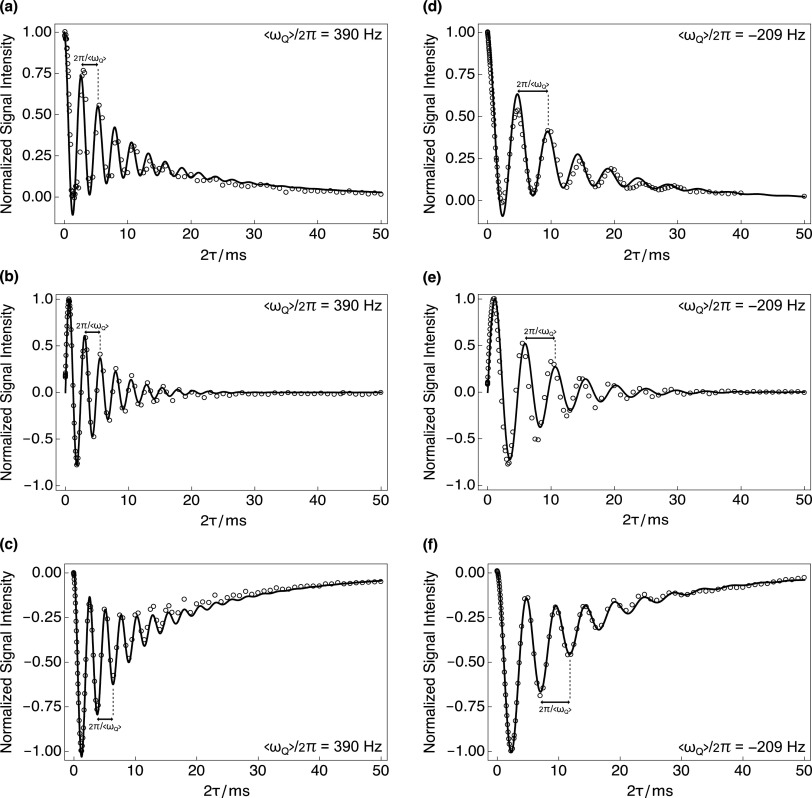
(a), (d) Rank-1, (b), (e) rank-2, and (c), (f) rank-3 SQC evolution trajectories of ^23^Na^+^ in uniformly stretched and compressed, respectively, hydrogel samples (extent = 15 cm) acquired using variants of the *rf*-pulse sequence shown in Fig. 2(a). Black empty circles: experimental data; black solid line: theoretical expression. Evolution trajectories were fitted using the following parameters: (a)–(c) $S_{0}^{*}$ = 4.6 × 10^−3^, *ω*_*Q*_/2*π* = 84.5 kHz, and *τ*_*C*_ = 1.72 ns; and (d)–(f) $S_{0}^{*}$ = −2.6 × 10^−3^, *ω*_*Q*_/2*π* = 81.5 kHz, and *τ*_*C*_ = 1.47 ns.

Experimental SQC evolution trajectories for ^23^Na^+^ in a uniformly stretched hydrogel sample are shown in Figs. 4(a)–4(c). The experimental data (black empty circles) are in good agreement with the solutions to the equations of motion (black solid curves) when using parameter values obtained from the analytical fitting procedure. The case of a uniformly compressed hydrogel sample is shown in Figs. 4(d)–4(f). Note that in Fig. 4, the overall sign (phase) of each evolution trajectory was unchanged by the action of stretching or compressing the gelatin-based sample.

Note that the experimental ^23^Na evolution trajectories for the rank-1 SQCs shown in Figs. 4(a) and 4(d) were not used as part of the numerical or analytical fitting procedure as the spin-echo experiment does not filter via coherence rank, which might cause problems for cell-based ^23^Na NMR experiments since isotropic populations of ^23^Na^+^ will not be filtered out. In these cases, the experimental data were directly overlain by the evaluated rank-1 analytical expression using the same set of best fit parameters, see Fig. S3 of the supplementary material. The agreement between experimental data and analytical expressions is remarkably good.

### F. Extent of stretching and compression

**FIG. 5.**
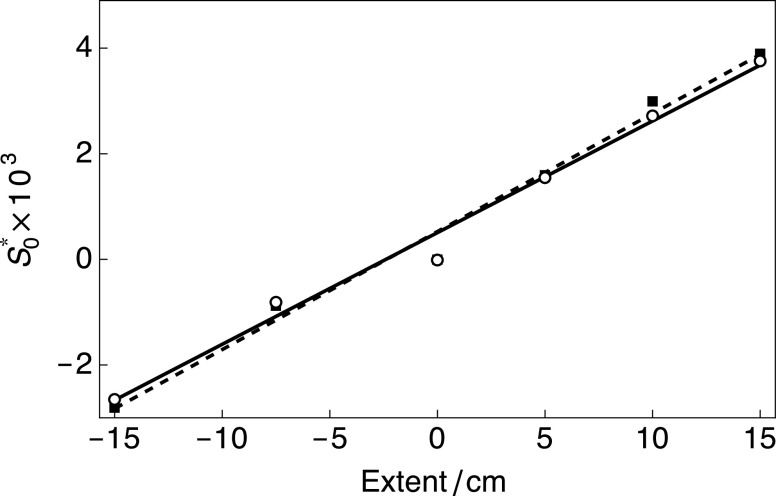
Spherical order parameters $S_{0}^{*}$ for ^23^Na^+^ in a hydrogel sample under several conditions of uniform stretching and compression and in the relaxed state. The data were fitted with a straight-line function including a nonzero intercept. Black empty circles and solid line: numerical values and best fit (slope = 0.21 × 10^−3^ cm^−1^, intercept = 0.50 × 10^−3^); black filled squares and dashed line: analytical values and best fit (slope = 0.22 × 10^−3^ cm^−1^, intercept = 0.52 × 10^−3^).

Figure 5 shows the spherical order parameter $S_{0}^{*}$ of ^23^Na^+^ in the hydrogel samples under several conditions of uniform stretching and compression and in the relaxed state. Numerically calculated values of $S_{0}^{*}$ varied from 3.77 × 10^−3^ (stretching extent = 15 cm) to −2.64 × 10^−3^ (compression extent = 15 cm). The data were fitted well by a straight-line function with a slope of 0.21 × 10^−3^ cm^−1^ including an intercept of 0.50 × 10^−3^. Similar values were found when fitting the experimental ^23^Na rank-2 and rank-3 trajectories to the analytical solutions of the equations of motion for SQC evolution derived in Sec. II B 1.

## V. DISCUSSION

### A. ^23^Na spectra

The experimental ^23^Na NMR spectra display the classical line shape for ^23^Na nuclear spins in the solution-state where alignment of the sample gives an RQC that manifests as a characteristic 3:4:3 splitting. Similar line shapes are observed in the ^71^Ga NMR spectroscopy of a semiconductor heterostructure^31^ and buried GaAs interface (partially resolved).^32^ A comparable (same order of magnitude) value of $S_{0}^{*}$ was previously determined for the ^17^O-enriched water–endofullerene H_2_O@C_60_ trapped within an aligned liquid crystal matrix.^20^

Experimental ^23^Na NMR spectra acquired under conditions of stretching or compression displayed outer transitions that were notably broader (shorter *T*_2_) than the central transition (longer *T*_2_). This is a consequence of intermediate to slow rotational reorientation, which leads to enhanced quadrupolar relaxation for these coherences relative to the central transition. The anisotropy of *T*_1_ values across the ^23^Na NMR spectrum is attributed to a buildup of rank-3 spin order ${\hat{\text{T}}}_{30}$ during the inversion recovery experiment, as has been previously reported in the literature.^21^

Spectral asymmetries of the outer peaks were attributed to quadrupolar-paramagnetic cross-correlated relaxation phenomena^33^ and a superposition of ^23^Na NMR spectra with a range of RQCs caused by the hydrogel pulling away from the Delrin plug, i.e., a gelatin sample with several extents of stretching. Similarly distorted line shapes have been observed for ^7^Li^+^ nuclear spins in stretched polystyrene gels.^34^

### B. Quadrupolar interaction

Analytical expressions are often sought to describe the time evolution of spin systems, including coherent evolution of the Hamiltonian and relaxation phenomena. The Hamiltonian that describes the evolution of the energy distribution between nuclear spin states in ^23^Na^+^ in an anisotropic medium (in which there is an EFG tensor that is aligned preferentially in the direction of **B**_0_) is well known and was used in the present work. There is both a secular term and a term that describes relaxation effects, as indicated by Eq. (3). As spin systems and pulse sequences become more complicated analytical expressions become unwieldy. Software packages such as *SpinDynamica*^35^ (written in *Mathematica*) can achieve outstanding outcomes that are well beyond by-hand derivations.

We derived solutions to the full equations of motion using a Liouvillian superoperator approach for ^23^Na^+^ (*I* = 3/2), which includes the coherent quadrupolar interaction and incoherent relaxation, to yield a complete analytical description for the evolution trajectories of rank-1 (${\hat{T}}_{1\pm 1}$), rank-2 (${\hat{T}}_{2\pm 1}$), and rank-3 (${\hat{T}}_{3\pm 1}$) SQCs observed in MQF NMR experiments performed on ^23^Na^+^ in mechanically aligned hydrogels. Numerical integration and matrix manipulation were implemented in *Spinach*. Overall, we have shown that both approaches are completely concordant not only for spectral line shape and time course simulations but also in fitting analytical expressions to data and output of numerical simulations. Both approaches gave the same parameter estimates in the relaxation analyses of experimental data from ^23^Na^+^ in stretched, compressed, and relaxed hydrogels.

### C. Microviscosity

The dominant quadrupolar relaxation mechanism of ^23^Na^+^ in partially aligned media is the result of fluctuating interactions between the electric quadrupole moment of the nucleus and EFGs at the nucleus. The rate of nuclear spin relaxation is dependent on the rotational correlation time *τ*_*C*_, which is determined by the microviscosity of the medium in which the ions diffuse.

An understanding of the factors that influence rates of enzymatic reactions between metabolites and transmembrane exchange of solutes has been provided by NMR spectroscopy. The iconic experiment by London *et al.* involved feeding ^13^C-labeled histidine to mice and extracting red blood cells that contained ^13^C-labeled hemoglobin.^36^ ^13^C NMR analysis of the values of the nuclear Overhauser effect and longitudinal nuclear spin relaxation time constants yielded estimates of *τ*_*C*_ of the hemoglobin molecules; remarkably, this was accomplished with fully intact cells. Later studies with ^13^C-labeled glycine and glutathione in human red blood cells provided additional insights into small molecule mobility inside these cells and led to the conclusion that no biochemical reactions are likely to be diffusion controlled.^37^

Our current experiments similarly provide an estimate of *τ*_*C*_ of the (hydrated) ^23^Na^+^ ion embedded within a hydrogel system. When inserted into the Debye equation, the mean-effective Stokes radius *a* of the ^23^Na^+^ ions can be computed from:

$$\tau_{C}=\frac{4\pi \eta a^{3}}{3k_{B}T},$$ (27)

where *η* is the microviscosity (assumed to be 1.7 × 10^−9^ Pa s, i.e., that of a haemolysate^38^) and *T* is the sample temperature. A rotational correlation time of *τ*_*C*_ ≃ 1.5 ns was measured for ^23^Na^+^ in a 35% *w*/*v* gelatin hydrogel system, which leads to a calculated Stokes radius of 0.95 nm. The ionic radius of ^23^Na^+^ found in a crystal, i.e., without its hydration shell, is ∼0.1 nm, while the Stokes radius in free solution is ∼0.25 nm, i.e., with its hydration shell.^39^ The much larger hydrodynamic radius in hydrogels therefore reflects the hindered rotation of the hydrated ^23^Na^+^ ion and its interaction with collagen molecules. Nevertheless, the values found for *τ*_*C*_ and *ω*_*Q*_/2*π* are reasonable for such hydrogel systems, with nearly identical values found previously for similar gelatin systems.^40^

The values of *ω*_*Q*_/2*π* are smaller than those previously reported for ^23^Na^+^ in other systems in the literature. Kuchel *et al.* reported *ω*_*Q*_/2*π* = 764 kHz in a glycerol/saline solution leading to *τ*_*C*_ = 7.48 ps,^41^ while Jerschow *et al.* quote a much larger value of *ω*_*Q*_/2*π* = 3.16 MHz in an aqueous electrolyte solution indicating that *τ*_*C*_ = 0.4 ps.^42^

Despite varying degrees of hydrogel anisotropy due to stretching and compression and in the relaxed state, we found very similar values for *ω*_*Q*_/2*π* and *τ*_*C*_ across all samples. These parameters are, therefore, largely independent of the anisotropic ordering of the gelatin matrix. Only the anisotropic contribution to the average RQC varied as a function of the degree of uniform hydrogel stretching or compression.

### D. RQC sign

The sign (phase) of $\left\langle \omega_{Q}\right\rangle$/2*π* depends on the statistical properties of ^23^Na^+^ orientation and is a consequence of the dependence of the RQC interaction on the Euler angle *β*_ML_ in Eq. (11a), which is either 0 or *π*/2 under uniform hydrogel stretching or compression, respectively. In the case of a uniformly stretched hydrogel, Eq. (10) indicates that motional averaging yields a net alignment of quadrupolar nuclear spins with the stretching direction (usually parallel to the **B**_0_-field axis, i.e., *β*_ML_ → 0), leading to a positive sign of $\left\langle \omega_{Q}\right\rangle$/2*π*. However, for uniformly compressed hydrogels, the overall alignment of quadrupolar nuclear spins is perpendicular to the direction of compression (and the **B**_0_-field direction) after motional averaging, i.e., *β*_ML_ → *π*/2, and Eq. (10) reveals that $\left\langle \omega_{Q}\right\rangle$/2*π* manifests a negative value. In our case, if $ArcTan\left[\sqrt{2}\right]<\beta_{\text{ML}}\leq \pi /2$, then Eq. (10) specifies $\left\langle \omega_{Q}\right\rangle /2\pi <0$, whereas $0\leq \beta_{\text{ML}}<ArcTan\left[\sqrt{2}\right]$ produces $\left\langle \omega_{Q}\right\rangle /2\pi >0$. Similarly, since $\left\langle \omega_{Q}\right\rangle =\omega_{Q}S_{0}^{*}$, the order parameter $S_{0}^{*}$ becomes negative once $\beta_{\text{ML}}>ArcTan\left[\sqrt{2}\right]$, but it is positive if $\beta_{\text{ML}}<ArcTan\left[\sqrt{2}\right]$.

### E. Gelatin content

Experiments were also conducted on a hydrogel sample with 60% *w*/*v* gelatin content but otherwise identical ^23^Na^+^ concentration, see Fig. S4 of the supplementary material. Figures S4(a)–S4(c) show NMR, DQF, and TQF spectra, respectively, for ^23^Na^+^ in a uniformly stretched hydrogel (extent = 15 cm). The spectra display an RQC of $\left\langle \omega_{Q}\right\rangle /2\pi =538$ Hz. It is interesting to note that this RQC is larger than portrayed in Fig. 3(b) for a lower gelatin concentration under an identical degree of stretching, which is indicated by the larger order parameter $S_{0}^{*}=4.8\times 10^{-3}$ for the stiffer gel.

Figures S4(d)–S4(f) show the corresponding SQC evolution trajectories. The experimental data are in agreement with the solutions of the equations of motion derived in Sec. II B 1. The evolution trajectories decay more quickly than those reported in Figs. 4(a)–4(c). The intrinsic value of *τ*_*C*_ was the same (to a good level of approximation) for samples with 35% and 60% *w*/*v* gelatin content. However, *ω*_*Q*_/2*π* was larger for the sample with increased gelatin concentration. The quadrupolar coupling frequency of ^23^Na^+^ will be highly dependent on the gelatin concentration, and owing to an increased number of interactions with the fibers of gelatin the rate of relaxation is increased.

### F. Sample reproducibility

The interactions between the electric quadrupole moment of the ^23^Na^+^ nucleus and EFGs at the nucleus arising from denatured collagen molecules, which make up gelatin, generate highly reproducible ^23^Na NMR spectral features from this particular gel. The commercial production of gelatin is very refined (for its extensive use as a food additive), thus minimizing inter-batch variation, which should facilitate reproduction of our present findings by different laboratories. This reproducibility of sample conditions was an important aspect of being able to repeatedly replicate our own ^23^Na NMR spectral outcomes.

After due attention to pH adjustment and correcting for the osmotic (oncotic) effect of concentrated protein solutions, gelatin is an ideal medium in which cells can be suspended and even grown. Its intrinsic strength and Hookean spring-like characteristics (up to a strain threshold for typically used concentrations that is a factor of two of the original length) make it a suitable medium for studying the effect of shape change on metabolic rate and cation transport, e.g., in red blood cells.^43^

### G. Biological applications

In skeletal muscle cells, the ^23^Na^+^ gradient across the sarcolemmal membrane is ∼1:10 (low concentration inside the cell). Generally, ^23^Na^+^ homeostasis is known to be dysregulated in cells in several disease states where it is elevated inside the cells;^44^ such states include ischemia, hypoxia, diabetic cardiomyopathies, heart failure, hypertrophy, and proliferative diseases, such as cancer. Methods that can measure such disturbances in cellular systems, *in vivo*, and clinically are of great current interest. ^23^Na NMR and MRI are the only techniques able to do this noninvasively in living systems. Ordered, systematically deformable media such a gelatin gels provide experimental models for refining NMR spectral approaches that can be applied to quasi-ordered tissues such as skeletal and cardiac muscle. It is often expedient to refine NMR approaches (and insights) with a well-defined and reproducible gel system rather than initially working with the more variable real tissues.

An example of this approach is our current quest for detecting ^23^Na^+^ pool sizes inside and outside cells with minimal chemical perturbation, that occurs with shift reagents;^11^ exploiting MQF NMR *rf*-pulse sequences such as are reported here. If the *τ*_*C*_ of ^23^Na^+^ differ inside and outside cells in suspensions, this can form the basis of discriminating relative population sizes. As such, this enables the noninvasive detection of migration (membrane transport) between the two populations without the use of a shift reagent to separate the intracellular and extracellular signals. NaK-ATPase is a major sink of adenosine triphosphate (ATP) free energy in cells, including red blood cells; hence, the activity of this ion pump is of interest to molecular cell biologists who study the energy economy of cells.^45^ Mathematical analysis of the NMR experimental results reported here, based on an almost ideal model system, provides one avenue to understanding the ^23^Na^+^ economy in whole cells and tissues.

## VI. CONCLUSIONS

We have demonstrated that a clearly observable RQC appears as an oscillation at a beat frequency of $\left\langle \omega_{Q}\right\rangle /2\pi$ in the experimental SQC evolution trajectories of guest ^23^Na^+^ in uniformly stretched and compressed hydrogel host systems. Numerical simulations and theoretical solutions to the equations of motion derived using a Liouvillian superoperator formalism describe the dynamics of the spin system remarkably well. Similar behavior is likely to occur for quadrupolar nuclear spins such as ^2^H and ^6^Li (*I* = 1), ^7^Li and ^39^K (*I* = 3/2), ^17^O and ^25^Mg (*I* = 5/2), and ^43^Ca and ^133^Cs (*I* = 7/2). This will be the subject of further investigation. These findings will facilitate exploration of ^23^Na^+^ properties in cells, e.g., red blood cells, cancer cells, or environments of interest in experimental and clinical MRI.

## SUPPLEMENTARY MATERIAL

The supplementary material related to this article is available online at: https://www.scitation.org/doi/suppl/10.1063/5.0158608. The supplementary material includes details of analytical expressions vs numerical simulations, numerical fitting, analytical fitting, and increased gelatin concentration.

## Data Availability

The data that support the findings of this study are available from the corresponding author upon reasonable request.

## NOMENCLATURE

DQF: double quantum filter(ed)
EFG: electric field gradient
FID: free induction decay
FT: Fourier transform
MQC: multiple quantum coherence
MQF: multiple quantum filter(ed)
MRI: magnetic resonance imaging
NMR: nuclear magnetic resonance
RF: radiofrequency
RQC: residual quadrupolar coupling
SQC: single quantum coherence
TQF: triple quantum filter(ed)
